# “It’s None of Their Damn Business”: Privacy and Disclosure Control in the U.S. Census, 1790–2020

**DOI:** 10.1111/padr.12580

**Published:** 2023-07-24

**Authors:** STEVEN RUGGLES, DIANA L. MAGNUSON

**Affiliations:** Institute for Social Research and Data Innovation, University of Minnesota, Minneapolis, 55455, USA.

## Abstract

The U.S. Census has grappled with public concerns about privacy since the first enumeration in 1790. Beginning in the mid-nineteenth century, census officials began responding to concerns about privacy with promises of confidentiality. In recent years, escalating concerns about confidentiality have threatened to reduce the usability of publicly accessible population data. This paper traces the history of privacy and disclosure control since 1790. We argue that controlling public access to census information has never been an effective response to public concerns about government intrusion. We conclude that the Census Bureau should weigh the costs of curtailing access to reliable data against realistic measures of the benefit of new approaches to disclosure control.

## Introduction

In response to concerns about privacy and government intrusion over the past two centuries, census officials have made promises of confidentiality. We argue that those promises have been ineffectual because privacy and confidentiality are not the same thing. We define privacy as individuals’ control over the transfer of information about themselves to others. Confidentiality, by contrast, is the promise by the U.S. government to prevent public disclosure of personal information (cf. Singer, Mathiowetz, and Couper 1993). When critics say, “it’s none of your damn business,” census officials respond with “we will keep it a secret from outsiders.” We argue that from the outset the critics of the census have been mainly concerned about government prying and respondent burden; the confidentiality promises to keep personal information within the government do not address those concerns.

We begin by describing the heated debate over the scope and content of the first census in 1790, the expansion of the census over the next five decades, and the first promises of confidentiality in 1840. The second section traces the increasing control of confidentiality breaches and data sharing across government entities between 1840 and 1950. We then turn to a systematic analysis of public discourse on census privacy and confidentiality and identify three great waves of concern in 1940, 1970, and 2000. Finally, we address disclosure control in census statistical publications since 1909, culminating with the Census Bureau’s adoption of formal privacy standards for the 2020 census. We conclude with a discussion of the implications of the history of privacy and disclosure control for current debates about access to data.

### The open census 1790–1840

#### The 1790 debate

The congressional debate over the content of the 1790 Census reflected concerns about government prying and respondent burden that have echoed over the past two centuries. James Madison, the chief designer of the first census, had originally proposed two census schedules. The first schedule focused on basic demographic questions: name of household head, white men age 16+, white men under 16, white women, slaves, and “all other persons” (free Blacks). The second proposed schedule focused on occupational questions, “specifying the number of persons employed in different professions and arts,” including merchants, mechanics, and manufacturers (Hobson and Rutland 1981, 8–9, 16; Gales 1834, 1077–1078, 1106–1109). Occupational data were necessary, Madison believed, because it provided “an opportunity for marking the progress of society and the growth of every interest” (Hobson and Rutland 1981, 8–9). Writing to then secretary of state Thomas Jefferson that the information in the occupational schedule was needed both for policy formation and for social science, Madison argued that repeating the questions every decade resulted in “a curious and instructive assemblage of facts” (Madison 1790).

The issue was hotly debated (Magnuson 1995). Representative John Page of Virginia worried that people would think the occupation questions were included merely for the “gratification of idle curiosity.” Senator Livermore of New Hampshire felt that such a scheme would potentially “excit[e] the jealousy of the people” and that citizens would fear its use for taxation and refuse to answer the census. Senator William Maclay of Pennsylvania, for his part, objected to the whole lengthy schedule because of the extra expense and the burden on respondents. In the end, the occupational questions were thrown out by the Senate. As James Madison wrote to Thomas Jefferson, Congress considered them a “waste of trouble and supplying materials for idle people to make a book” (Gales 1834, 1114–1115, 1145–1147; Maclay 1890, 195; Madison 1790).

The core concerns in the debate over the 1790 census recurred again and again over the next 23 censuses. Those in favor of expanding the census argued that the information was needed for “adapt[ing] the public measures to the particular circumstances of the community” and “marking the progress of the society” (Congressional Register 1790, 167–168). Critics countered those additional questions by the government as too intrusive and too expensive. There was particular concern that the public suspected that the information would be used against them, especially for taxation. For example, the *Aurora General Adviser*, published in Philadelphia in November 1790, suggested that smaller and poorer families must have “tempted many to conceal” their numbers, for fear of taxation (“Philadelphia,” 1790).

In July of 1791, President Washington wrote in a letter to Gouverneur Morris regarding the disappointing 3.9 million persons enumerated in the first census “that the real number will greatly exceed the official return; because, from religious scruples, some would not give in their lists; from an apprehension that it was intended as the foundation of a tax, others concealed or diminished theirs…” (Washington 1791a). Some assistant marshals reported that they encountered refusals by heads of households “to Render an Account of his family pursuant to the directions of the aforesaid Act” (Washington 1791b). One assistant marshal reported a “prodigious deal of trouble” in obtaining information from respondents (Fliss 2000, 87; Washington 1791b).

### Census expansion and the origins of confidentiality, 1800–1840

After 1790, advocates for more detailed censuses continued to press their case. In 1800, Thomas Jefferson proposed additional questions on occupations, detailed age, birthplace, and citizenship (Jefferson 1800). The president of the Connecticut Academy of Arts and Sciences Timothy Dwight, also advocated for data on age groups, occupations, and marital status. In 1830, Congressman Charles F. Mercer “was in favor of giving a still greater extent to the subjects required to be enumerated and returned by the marshals…” (Gales and Seaton 1855, 922–923). President John Quincy Adams used his fourth annual message to congress as an opportunity to smooth the way for expanding the age categories on the 1830 census: “the result would exhibit comparative tables of longevity highly interesting to the country” (Adams 1828). Martin Van Buren also used his presidential annual message to suggest “extending” the census:

In recommending to Congress the adoption of the necessary provisions at this session for taking the next census or enumeration of the inhabitants of the United States, the suggestion presents itself whether the scope of the measure might not be usefully extended by causing it to embrace authentic statistical returns of the great interests specially intrusted [sic] to or necessarily affected by the legislation of Congress. (Van Buren 1838)

Although the advocates for census expansion did not get everything they asked for, the number of questions on the population schedule expanded from six in 1790 to 80 in 1840.

There were no promises of confidentiality of the population schedule before 1850. The 1790 Census Act specified that upon completing their enumeration of a district, each Assistant Marshall shall “cause a correct copy” of the census returns “to be set up at two of the most public places” in his district, “there to remain for the inspection of all concerned” (Census Act 1790; Wright and Hunt 1900; Eckler 1972). The idea was that copies of the census returns posted in the local post office or tavern would enable members of the public to spot errors or omissions in the enumeration. The length of time for public inspection was not specified, “but must be presumed a reasonable time … within which all the inhabitants recorded in the schedule may have had a sufficient opportunity for the inspection thus offered them … ” (Wright and Hunt 1900).

Confidentiality promises first emerged not because of the questions on the population schedule, but rather, with the questions on the census of manufactures. In 1810, James Madison—by then the President—finally got the separate manufacturing schedule he had first proposed in 1790 (Wright and Hunt 1900). Assistant marshals encountered resistance to providing information about business operations because a “rumor circulated” in advance of the canvass that “the information was required for tax purposes” (Fishbein 1973, 3). In 1820, the census of manufactures was compromised by the “reluctance of certain manufacturers to supply data” (Fishbein 1973, 11). Some assistant marshals recorded respondent objections “directly on the schedules” and “in other cases the deputy marshals noted that the proprietors were either fearful of taxation or angry at the invasion of their privacy” (Fishbein 1973, 11). For example, assistant Marshal Abijah Smith in Maine wrote at the end of his returns: “The answers given to Questions No. 9 and 13, were not to me perfectly satisfactory. Thro [sic] fear of an increase of Taxes, or from some other cause, they could not be prevailed upon to make their answers more definite” (U.S. Census Office 1820a). John Langdon, also an assistant marshal in Maine, observed that “This part of my duty was attended with some difficulty as the Individuals generally suspected it was preparatory to Taxation” (U.S. Census Office 1820b). In Massachusetts, several manufacturers refused to provide information for unspecified reasons (U.S. Census Office 1820c, 1820d, 1820e).

No census of manufacturing was attempted in 1830. Manufacturing questions at the census of 1840 were part of a “Schedule of Mines, Agriculture, Commerce, Manufactures, etc.” (Wright and Hunt 1900). There were 79 agricultural questions, including questions on beeswax and silk cocoons. Virginian John Hampden Pleasants complained about the agricultural census in a letter to the editor reprinted in several newspapers: “Is this Federal prying into the domestic economy of the People a precursor to *direct taxes?* Is nothing to escape its inquisition or its tax-gatherers? Are even our hens and chickens to be listed, and an authenticated *expose* forwarded to Washington?” (Pleasants 1840).

For the first time in 1840, the Census Office responded to concerns about government intrusion with guarantees of confidentiality. The 1840 instructions to U.S. Marshals for collecting “all such information in relation to mines, agriculture, commerce, manufactures, and schools” noted that “Objections, it has been suggested, may possibly arise on the part of some person to give the statistical information required by the act, upon the ground of disinclination to expose their private affairs.” And for the first time, the instructions made a promise of disclosure control, noting that in the published tables “no name is inserted—the figures stand opposite no man’s name.” Assistant marshals were instructed to “consider all communications made to him in the performance of this duty, relative to the business of the people, as strictly confidential.” This new confidentiality policy applied only to the schedule covering mines, manufacturing, commerce, and agriculture; the 80 questions of describing the characteristics of the population were still publicly posted in prominent places to allow the public to make corrections (Wright and Hunt 1900).

### Limits on the gratification of curiosity, 1840–1950

#### Prohibition of confidentiality breaches, 1850–1900

The 1790 directive to post the manuscript population schedules in a public place for “the inspection of all concerned” to facilitate verifying and correcting returns was discontinued in 1850, and, instead, a copy of the returns was deposited with their respective county clerk (Bohme and Pemberton 1991). This change may have partly reflected a major redesign of the census population schedule. Instead of recording summary statistics for each household, the 1850 census collected information on each individual. These included potentially sensitive questions on the value of property, birthplace, literacy, detailed occupation, criminal conviction, and disabilities.

For the first time, the 1850 enumeration instructions admonished that personal information was to be kept confidential. Marshals were specifically directed in a circular “to consider the facts intrusted [sic] to them as if obtained exclusively for the use of the Government, and not to be used in any way to the gratification of curiosity, the exposure of any man’s business or pursuits, or for the private emolument of the marshal or assistants.” The Census Office had received information that “in some cases unnecessary exposure” had been made by assistant marshals “with reference to the business and pursuits, and other facts relating to individuals, merely to gratify curiosity” (Wright and Hunt 1900, 150).

The general instructions to assistant marshals repeated this guidance in 1860 and noted that in 1850 “Cause for offense was given by one or two indiscreet assistants … by the liberty exercised in the unnecessary exposure of facts relating to the business and pursuits of individuals … to persons who desired it for private advantage or pecuniary profit, or to newspapers” (U.S Census Office, 1860, 12). The language of expected confidentiality on the part of canvassers was strengthened in 1870:

No graver offense can be committed by assistant marshals than to divulge information acquired in the discharge of their duty. All disclosures should be treated as strictly confidential, with the exception hereafter to be noted in the case of the mortality schedule. Information will be solicited of any breach of confidence on the part of assistant marshals. The department is determined to protect the citizen of all his rights in the present census. (Wright and Hunt 1900, 156)

Congressman (later president) James A. Garfield advocated at the 1870 census for a statutory penalty for census data disclosure because “the citizen is not adequately protected from the danger, or rather the apprehension, that his private affairs, the secrets of his family and his business, will be disclosed to his neighbors” or “made the quarry of bookmakers or pamphleteers” (“Right to Privacy” 1981, 1905).

In 1880, the census made it a misdemeanor with a penalty up to $500 for “communicat[ing], without the authority of the Superintendent of Census, to any unauthorized person any statistics of property or business included in his return” (Wright and Hunt 1900, 66). An entire section with the heading, “PENALTY FOR DISCLOSING INFORMATION,” was included in the 1890 instructions to enumerators: “The intent of this provision is to make the answers to all the inquiries confidential, and to prevent disclosures of information which would operate to the personal detriment or disadvantage of the person supplying the same” (U.S. Census Office 1890, 6; Wright and Hunt 1900). This language was repeated in the Census Act of 1900 (Wright and Hunt 1900; Eckler 1972).

### Data sharing across government entities

Despite the increasing controls on disclosure of census information after 1850, census information was widely shared across government agencies and sometimes with nongovernmental entities. In 1870 Superintendent of the Census Francis A. Walker criticized the practice of depositing a copy of the census returns with county offices on the grounds that this posed an intolerable threat to confidentiality. He wrote

It is useless to attempt to maintain the confidential character of a census under such circumstances. The deposit of the returns at the county seat of every county constitutes a direct invitation to impertinent or malicious examination. No proper purpose can be served by this copy of the census returns. All the use to which it can be put must be improper and mischievous. At every step the work of the assistant marshal has been made more difficult by the fear that the information would be used with a view to taxation, or that matters strictly of family or personal interest would be divulged for impertinent and malicious criticism. (Walker 1871, xxiii)

Access to population schedules by state and municipal governments was explicitly permitted (for a modest fee) beginning in 1890.

That upon request of any municipal government … the Superintendent of Census shall furnish such government with a copy of the names, with age, sex, birthplace, and color or race, of all persons enumerated within the territory in the jurisdiction of such municipality, and such copies shall be paid for by such municipal government. (Wright and Hunt 1900, 947–948, 955–956)^1^

The 1910 census act extended access to population schedules at the “discretion” of the Director of the Census to include individuals seeking data “for genealogical or other purposes” (Census Act 1909, 10).

Over subsequent decades, access was gradually tightened (Department of Commerce and Labor 1913, 15–18). The act authorizing the 1920 Census specified that “Director of the Census [shall not] permit anyone other than the sworn employees of the Census Office to examine the individual reports.” A decade later, the Census Bureau further clarified that the 1929 Census Act protected the information collected from being used “to the detriment of the person or persons to whom such information relates” (Census Act 1919, 13; Census Act 1929, 26).

In 1910, President Taft had issued the first presidential proclamation regarding the census and in it he promised that “There need be no fear that any disclosure will be made regarding any individual person or his affairs” (Taft 1910). The tension between public promises of confidentiality and intragovernmental expectations for access to census data accelerated when the United States entered the Great War in 1917. There was widespread opposition to American entry into the war, and resistance to conscription was significant. Census Director Samuel L. Rogers believed that “every branch of Government, including this Bureau, should assist … as far as possible, in securing a full [draft] registration” (Anderson 2015, 129). Thus, the Department of Justice, in its effort to enforce compliance with the Selective Service Act (1917), requested and received information to investigate draft evasion and figures to estimate eligible numbers of draftees (Bohme and Pemberton 1991, 10–11; Anderson 2015, 129–130). In peacetime in 1930, the Women’s Bureau requested a list of names, addresses, occupations, and employment status of women in Rochester, New York. The Census Bureau referred the request to the Attorney General, who ruled against disclosing the requested information (Eckler 1972, 168).

The Second World War sparked another wave of intragovernmental sharing of census information. The passage of the Second War Powers Act (1942) stated that the Secretary of Commerce, at the discretion of the President, was permitted to “make such special investigations and reports of census or statistical matters as may be needed in connection with the conduct of the war” (Second War Powers Act 1942, 186). Thus, census data could be used by any branch of the government for war purposes (Roosevelt 1942; Anderson 2015). Census Director J.C. Capt was unflinchingly committed to providing census data as necessary to support the war effort (Anderson 2015). Seltzer and Anderson (2007) documented multiple examples of wartime disclosure of individual-level census responses.

### Three waves of anxiety about census privacy, 1940–2000

#### Public discourse on privacy and disclosure control

**FIGURE 1:**
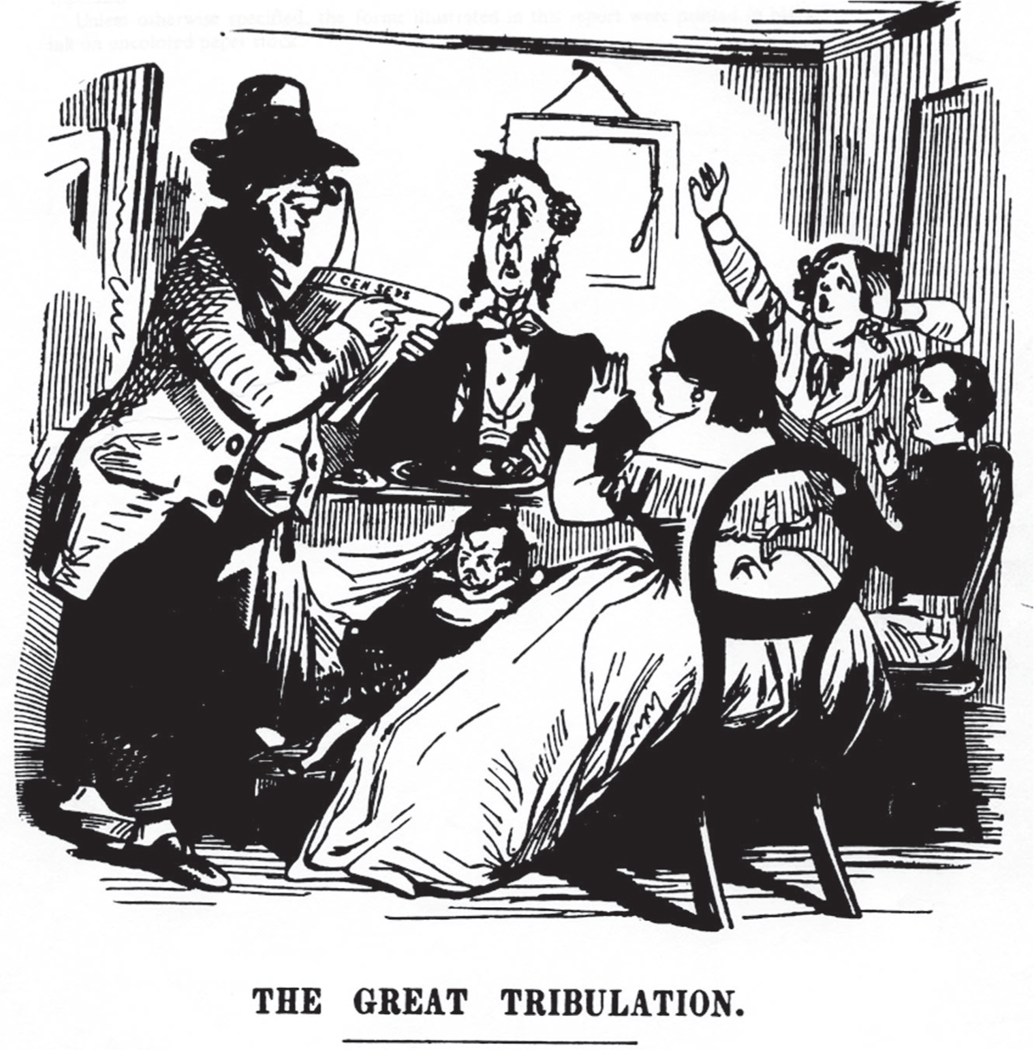
**Census Marshal.--** “I jist want to know how many of yez is deaf, dumb, blind, insane, and idiotic—like-wise how many convicts there is in the family—what all your ages are, especially the old woman and the young ladies—and how many dollars the old gentleman is worth!” [Tremendous sensation all round the table.] SOURCE: *Saturday Evening Post*, August 18, 1860.

To investigate trends in public anxiety about privacy in the census, we conducted a systematic analysis of newspaper articles. We identified 464 articles in 31 newspapers between 1790 and 2022 that referred to census privacy or confidentiality. We identified little public concern about census privacy before the twentieth century, just a few isolated newspaper cartoons and articles that poke fun at the intrusiveness of the census. Figure 1 shows a *Saturday Evening Post* cartoon published in 1860 featuring invasive questions that appeared on census forms from 1850 to 1870 (U.S. Census Bureau 1979, 6). In 1875, *The New York Times* described the census taker as “a busybody in other men’s matters” and “an impertinent spy” who “invades domestic privacy” (“Trials of the Census Taker” 1875). In 1880, an article in the *Arkansas Gazette* observed that the census enumerator was “Forced, by reason of duty, into the privacy of people’s houses and domestic affairs” and was therefore “browbeaten and snubbed and subjected to all manner of abuse …” (“The Census Takers’ Pay” 1880).

To obtain a reasonably comparable chronological series, we focused on 230 articles in four major newspapers that are digitally searchable back to 1881: *The Los Angeles Times*, *The Minneapolis-Star Tribune*, *The New York Times*, and *The Washington Post*. Each of these 230 articles identifies a concern about privacy or confidentiality of the census by a member of the public, a legislator, or some other entity. We classified these public concerns into four categories: (1) disclosure of census information to someone outside of government; (2) improper use by the government (e.g., enforce laws about taxes, a military draft, or immigration); (3) government invasion of privacy, or “Big Brother”; (4) concerns about the Census Bureau’s disclosure avoidance system, especially the adoption of the differential privacy criterion for the 2020 census.

**FIGURE 2:**
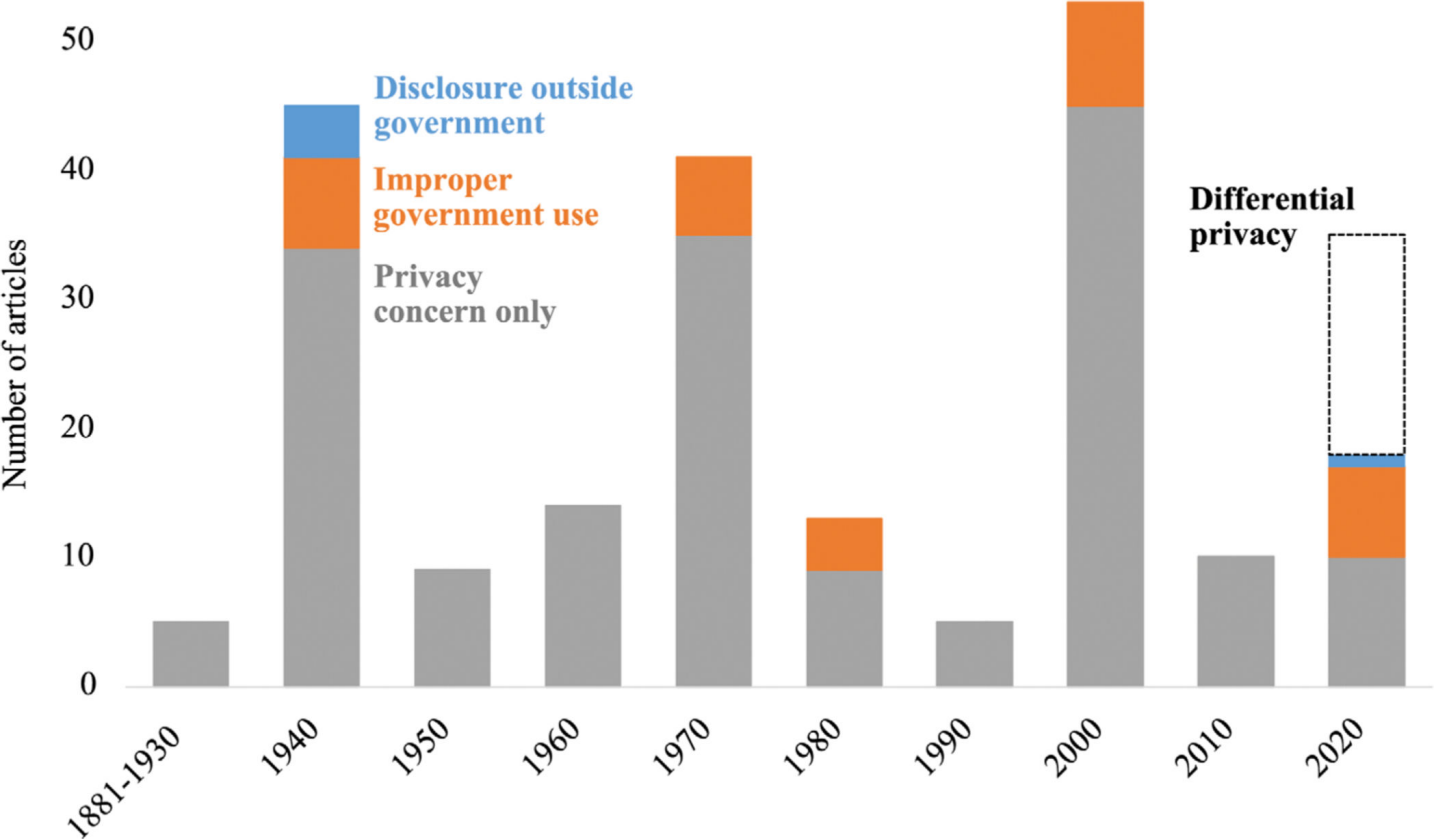
Newspaper articles discussing census privacy, by type of concern: *LA Times*, *Minneapolis Star and Tribune*, *NY Times*, *Washington Post*, 1870–2020

The number of articles in each category surrounding each census appears in Figure 2. Only four articles pertain to the period before 1940. There were three major waves of interest in census privacy, in 1940, 1970, and 2000. The great majority of articles were concerned about privacy, usually expressed as government intrusion. Only five of the 230 articles mentioned a risk of disclosure of census information to anyone outside of government. Four of these concerns were reported in 1940; the fifth expressed concern that the 2020 census could be vulnerable to hackers because the Census Bureau inadequate data security infrastructure (“The Cybersecurity 202” 2019). A few newspaper accounts scattered across the period from 1940 to 2020 expressed concern about misuse of the census data by other branches of government, such as the Internal Revenue Service (Krock 1940b; “Census and Privacy” 1969; Anthony 1970; “Census bill seeks wider access” 1977; “Some ‘weird’ views” 1978; “Censuses’ most invasive question” 2000; “Groups take aim” 2019).

Articles about privacy and disclosure in the 2020 census focused on two main themes. The Trump administration had attempted to add a question on citizenship to the census at the last moment, and this generated fears that the information might be used improperly by government agencies such as Immigration and Customs Enforcement. The second concern in 2020 was about the Census Bureau’s new approach to disclosure avoidance based on the concept of differential privacy. The Census Bureau argued that tabular data ordinarily published by the census could be attacked by outsiders to reveal protected census responses. The newspaper coverage related to differential privacy emanated entirely from within the Census Bureau and was not sparked by any public concern about census privacy, so we have represented them with a dotted bar in Figure 2. If we exclude articles about differential privacy, 2020 does not show exceptional concern with census privacy, despite the uproar about the citizenship question.

#### 1940: All the privacy of a gold fish

**FIGURE 3:**
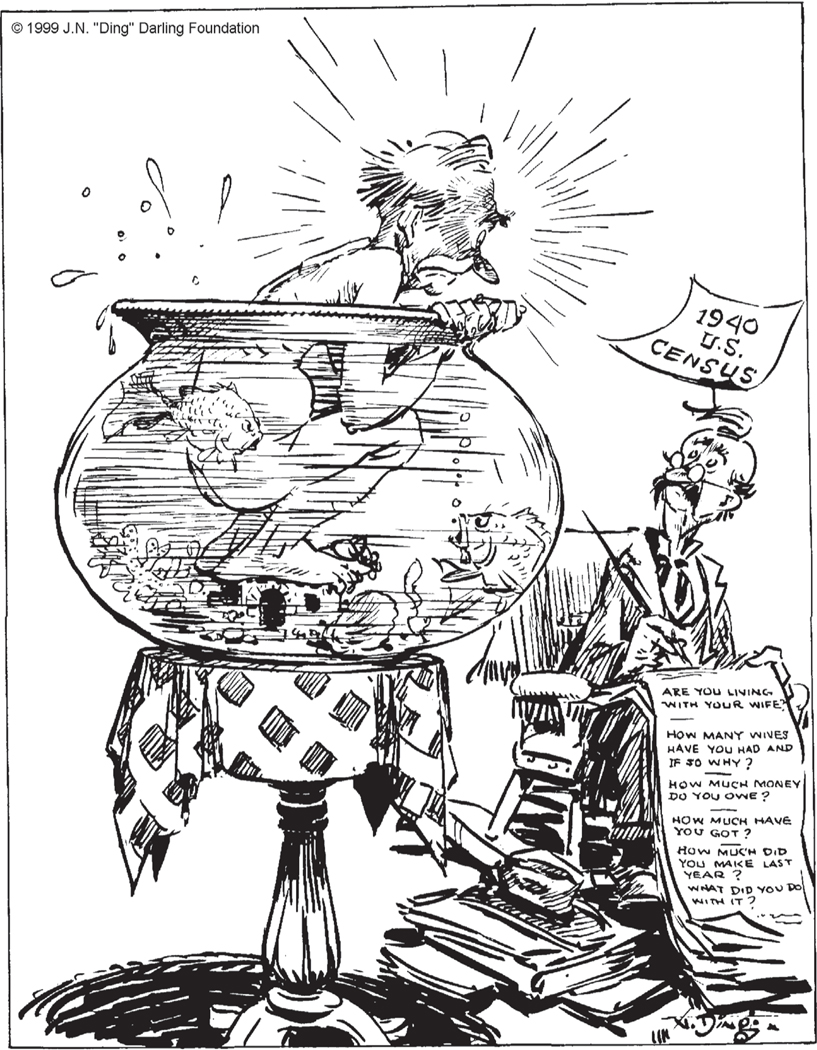
Text: 1940 U.S. Census. Are you living with your wife? How many wives have you had and if so why? How much money do you owe? How much have you got? How much did you make last year? What did you do with it? Reproduced by permission of the “Ding” Darling Wildlife Society, which owns the copyright to “Ding” Darling cartoons. SOURCE: *Minneapolis Star-Ledger*, February 25, 1940, p. 10.

The first wave of census anxiety occurred in 1940. The number of population and housing questions more than doubled from 30 in 1930 to 65 in 1940. For the first time, the census included a question on “Amount of money wages or salary received (including commissions)” (U.S. Census Bureau 2002, 59, 62–63). Even “pickpockets, burglars, and bandits” of Bridewell Prison in New York “considered this a violation of their right to privacy” (“Bridewell Inmates” 1940). The *Minneapolis-Star Ledger* captured the sense of invasion of privacy attributed to many Americans in the cartoon shown in Figure 3, “All the Privacy of a Gold Fish” (1940).^2^ Unlike the similar 1860 cartoon “The Great Tribulation,” the 1940 cartoon included questions that were not actually on the census form.

Senator Charles W. Tobey (R-NH) led a charge to eliminate the income question from the census.

I protested just as soon as the terrible violation of privacy came to my eyes … It isn’t necessary in a Republic such as ours, that every citizen should live in a goldfish bowl … There are just some things that are none of the public’s business. If the Government keeps on encroaching on the rights of its citizens, we’ll be no better off than our fellow human beings who live under despotic forms of government. (“Census: Are Questions on Income Legal” 1940)

Tobey used inflammatory language to characterize the income question as “inquisitorial” and pushed for removal of the question on the schedule (“Census Called Inquisitorial”1940).“You might as well ask questions about the use of contraceptives or about relations between husbands and wives” (“Predict Erasure of Income” 1940). When his approach to foment “rebellion” failed, Tobey urged concerned citizens to refuse to answer the “snooping” questions (“Senator Tobey” 1940; “Revolt on Census” 1940; “Warns Snoopers” 1940). Though President Roosevelt branded Tobey’s efforts as a “revolt,” he offered a “concession” to those who had privacy concerns about the income question. The Census Bureau devised a plan “by which persons who are unwilling to tell census enumerators the amount of their salaries, can forward the information anonymously to Washington” (“Roosevelt” 1940; “Hopkins” 1940; “Census Income Questions” 1940). Under this plan, those who refused to disclose their income directly to the enumerator were given a confidential form and an envelope addressed to the Census Director that they could complete in private. Only 200,000 respondents took this option, and another 1.5 million did not answer the income question at all (Jenkins 1983, 18, 37).^3^

The issue of privacy was the featured lead item in the *New Yorker’s* “Talk of the Town” column. The column opens:

BEYOND any doubt we are living in the great era of question-asking, the heyday of official curiosity. On April 2nd, the census takers, covering the land like locusts, are going to want to know more about us than they ever did before—more about our house, more about our income, more about where we were on the night of January 16th. We have been reading that a lot of statesmen are dismayed by this prospect, seeing their constituents’ privacy ruthlessly violated [this of course is a reference to Senator Tobey]. It is our opinion that this is largely waste motion … We don’t think the census-taker is going to have any special trouble getting the facts out of people. (“Talk of the Town” 1940)

#### 1970: The national data center and big brother

The second wave of census anxiety began in the 1960s and was fueled by concerns about “Big Brother.” The context surrounding this wave was the recommendation by the Committee on the Preservation and Use of Economic Data to the Social Science Research Council, to create a national data center. The idea behind the Data Center was to make data from different federal sources interoperable, preserve it, and make it accessible to researchers under strict disclosure guidelines (Ruggles 1965). The Johnson Administration supported the idea, and it appeared things were moving in the direction of the formation of the Data Center (Kraus 2013). Then abruptly, there was a backlash.

Congressman Cornelius Gallagher (D-NJ) led the charge, accusing the authors of the proposal of trying to create computerized dossiers on every American (Allen and Scott 1966). In a hearing before the Invasion of Privacy Subcommittee, Gallagher asserted that “The presence of these records in Government files is frightening enough, but the thought of them neatly bundled together into one compact package is appalling. We cannot be certain that such dossiers would always be used by benevolent people for benevolent purposes” (Gallagher 1966, 3). The *Atlantic* published an article by Arthur Miller, a civil procedure professor at the University of Michigan, who wrote that the Data Center

Poses a grave threat to individual freedom and privacy. With its insatiable appetite for information, its inability to forget anything that has been put into it, a central computer might become the heart of a government surveillance system that would lay bare our finances, our associations, or our mental and physical health to government inquisitors or even to casual observers. (Miller 1967)

The press picked up Gallagher’s concerns and hundreds of articles appeared in newspapers and magazines across the country (Kraus 2013, 13–17). Gallagher was quoted as saying, “People worry about who has the button on nuclear weapons. We’ve got to start worrying about who has the button on the computer” (Lardner 1966a). *The New York Times* ran a story with the headline, “Professor Warns of Robot Snooper: Tells Senators Data Bank Could Destroy Privacy.” Here too Professor Miller reportedly warned that “a computerized national data bank could become a monstrous ‘Big Brother’ with an insatiable appetite for snooping” (“Professor Warns” 1967, 36). The “all-seeing eye” was a “hallmark of totalitarianism” (Packard 1967). Witnesses at the hearings on the proposal for the National Data Center reportedly “assailed the plan” as a “threat to individual liberty” and a “harbinger of Big Brother, and a mechanized suffocation of the American dream” (Lardner: 1966b). Gallagher wrote that “More than ever before, man has the horrendous potential for creating his own version of hell—a computerized, dehumanized, unidimensional society, inhibited by no law or moral consideration in the exercise of its enormous power over all individuals” (Gallagher 1967, 113).

Why this wave of panic in the 1960s? Partly it was the fear of the computer—the “electronic brain,” which in science fiction often proved to have malevolent intent (Manning 1964, 191). Some fears, however, were realistic. For example, FBI Director J. Edgar Hoover’s abuses of power had been coming to light. It was an open secret that the FBI maintained files on millions of Americans that were sometimes used to intimidate and blackmail (Jeffreys-Jones 2007; Weiner 2012). The leap from “the electric brain” to “dossiers on every American” did not seem so far-fetched in this context.

By 1970, as the furor over the National Data Center and Big Brother seemed to be ebbing, it was revived by Republican Congressman Jackson Betts (OH). For 19 years, Betts was an obscure figure in the House of Representatives. Then, he stumbled on an issue: the “intrusive” questions contained in the 1970 census. Betts got abundant press coverage on the issue, but not much traction (Valentine 1968; “Curb on curiosity” 1967; “Plea to cut queries” 1967; “Will ‘70 Census Be Too Personal?” 1969; “Census Privacy” 1969). In the end, even Cornelius Gallagher—the congressman from New Jersey who led the charge against the National Data Center in the late 1960s, turned on Betts, and affirmed the “government’s need to know” (Quiet ‘Country Congressman’1969).

#### 2000: “It’s none of their damn business”

The third wave of census anxiety emerged from the convergence of the rise of talk radio and Republican politicians raising concerns about the census and privacy (Berry and Sobieraj 2011; Matzko 2020). The context of this wave lies in the decision of the Federal Communication Commission to dispense with the Fairness Doctrine in 1985, opening the airwaves to “ideologically based programming” (Berry and Sobieraj 2011; Matzko 2020; Barker 2002, 16). Talk radio “got louder” between the censuses of 1990 and 2000 (“Overly Overwrought About the 2000 Census” 2000; Barker 2002, 24). Right-wing radio hosts were free to fill drive-time with diatribes about government intrusion represented by the census long form (“Census Bashing” 2000; Hofstetter et al., 1999; Barker, 2002). Before the census was even underway in April 2000, “From newspapers to television, and from talk radio to congressional offices, everyone was talking about privacy and the perceived intrusive nature of the long form questionnaire” (Miller 2000; “Census Bashing,” 2000; Cohn 2000a).

Senator Chuck Hagel (R-NE), Senate Majority Leader Trent Lott (R-MS), and governor and presidential candidate George Bush (R-TX) were vocal in their objections to the census long form on privacy grounds (Cohn 2000b; “Census Bashing” 2000; “Census Nonsense” 2000; Casey 2000). Hagel said, “Just fill out what you need to fill out, and [not] anything you don’t feel comfortable with” (“Census Bashing” 2000). Lott’s “advice to his fellow Americans” was, if they “feel their privacy is being invaded by [some] questions, they can choose not to answer” (“Census Bashing” 2000). On the presidential campaign trail Bush said, he was not sure he would want to fill out the long form (“Trashing the Census” 2000).

Hagel, Lott, and Bush did not cite a risk of public disclosure; rather, in Hagel’s words, “I don’t know why the government needs all that information … It’s none of their damn business” (Wegner 2000). In Congressional testimony on April 3, 2000, Carolyn Bosher Maloney (D-NY) rebuked the men,

What is really amazing with this newfound concern about the census is that, over 2 years ago, really 3 years ago also, the content of the long and short forms and while it was being finalized, every single Member of the House of Representatives and the United States Senate received a detailed list of the questions to be asked, including a description of the need for asking it, along with the specific legal requirement supporting it. (Maloney, 2000)

Our analysis of newspapers found that in all three waves of concern about census privacy, the overwhelming concern was about invasion of privacy, not disclosure risk. As Hillygus et al. (2006, 122) noted, “When President Bush said in 2000 that if he got the long form, he was not sure he would answer all the questions, it was not fear of disclosure he had in mind but government intrusiveness.” There was a scattering of concern that the government might do something nefarious with the information. Concern about disclosure of census responses to someone outside the Census Bureau was exceedingly rare; we identified just five articles that expressed such fears, four of them from the period around the 1940 census (“Salary question” 1940; “Attack Continues” 1940; Krock 1940a, 13; Krock 1940b, 19; “Letter to the Times” 1940).

### Disclosure control for census publications

#### Legal changes, 1909–2002

Between 1850 and 1900, concerns about breaches of confidentiality focused mainly on the conduct of temporarily employed decennial canvassers. After the Census Office became a permanent agency in 1902, the newly formed Bureau of the Census expanded its oversight for disclosures beyond canvassers to include official publications. Section 25 of the 1909 Census Act specified that the information collected “shall be used only for the statistical purposes for which it is supplied.” Concern with disclosure in census publications was “particularly great” with the industrial census, “where a single large unit may dominate the total for a small population” (Eckler 1972, 166). The law specified that “No publication shall be made by the Census Office whereby the data furnished by any particular establishment can be identified” (Census Act 1909, 9). The 1929 census law was the first to extend the promise to individuals, specifying that “No publication shall be made by the Census Office whereby the data furnished by any particular establishment *or individual* can be identified” [emphasis added] (Census Act 1929, 25).

Virtually the same language appears in Title 13 (1954), which has governed the censuses taken since 1960. The statute requires that no officer or employee of the Census Bureau may “make any publication whereby the data furnished by any particular establishment or individual can be identified” (Title 13 U.S.C. § 9).

In 1962, Title 13 was amended to make it explicit that census returns could not be shared with other branches of government and could not be subpoenaed in legal proceedings:

No department, bureau, agency, officer, or employee of the Government, except the Secretary in carrying out the purposes of this title, shall require, for any reason, copies of census reports which have been retained by any such establishment or individual. Copies of census reports which have been so retained shall be immune from legal process, and shall not, without the consent of the individual or establishment concerned, be admitted as evidence or used for any purpose in any action, suit, or other judicial or administrative proceeding.

Bohme and Pemberton note that “The long-standing permission to furnish individual data as described above to governors of states and territories and to courts of record as well was not removed from the census law (Title 13 U.S.C. §8) until 1976” (Bohme and Pemberton 1991, 6).

The Confidential Information Protection and Statistical Efficiency Act of 2002 (2002) explicitly defined the concept of identifiable data: it is prohibited to publish “any representation of information that permits the identity of the respondent to whom the information applies to be reasonably inferred by either direct or indirect means” (Title 44 U.S.C. §3561 (7)). The Foundations for Evidence-Based Policymaking Act of 2018 (2019) reiterated the interpretation that the law is designed to protect respondent identities.

### Disclosure control in machine-readable data, 1960–2020

Until the early 1960s, census data were disseminated exclusively through printed volumes. This format imposed practical limits on the amount of detail presented and limited threats to confidentiality in census publications. In 1962, the Census Bureau released the first electronic publication of census data, providing individual-level records (or microdata) drawn from the 1960 “long form” census, a detailed survey filled out by one in four households. The documentation explained that the data were compliant with census privacy law:

The one-in-a-thousand sample makes available reels of magnetic tape or sets of punchcards containing the separate records of the characteristics of a 0.1 percent sample of the population of the United States as recorded in the 1960 census.Thenamesoftherespondentsandcertainmoredetaileditemsonplace of residence are not revealed. Therefore, it has been determined that making records available in this form does not violate the provision of confidentiality under which the census was conducted. (U.S. Census Bureau 1962, 2)

The release of individual-level information was not seen as a violation of Title 13 because the Bureau did not reveal the identity of individuals. In addition to removing names and addresses, the Census Bureau suppressed geographic detail below the state level and top-coded income to prevent the identification of high-income persons. Only one of every 1,000 persons was included in the sample, and there was no way for an outsider to determine whether any particular individual was represented.

In the mid-1960s the Census Bureau also began distributing summary tapes containing tabular data. These tapes included tables that the Census Bureau had prepared as an intermediate step in creating the 1960 census publications, providing more detail than was available in the printed census volumes (U.S. Census Bureau 1964, 1967a, 1967b). To protect confidentiality, the Census Bureau suppressed the data for geographic units with a very small population count (Courtland 1985).

The basic methods of privacy protection in Census Bureau data products remained essentially similar until 1980 for both microdata and tabular files, although the details varied by year. Public Use Microdata Samples (PUMS) were protected by (a) stripping off names and other identifying information, (b) providing only a sample of the original data, (c) suppressing detailed geographic information, (d) top-coding continuous variables such as income, and (e) collapsing some very detailed categories such as place of birth (Federal Committee on Statistical Methodology 2005). The tabular information was protected by suppressing the data in places with very small populations or with few members of particular subgroups (McKenna 2018).

In 1990, there was a major innovation, as described in the Census Bureau’s *Monograph on Confidentiality and Privacy*:

The data from [a sample of] households were swapped with data from other households that had identical characteristics on a certain set of key variables but were from different geographic locations. Which households were swapped was not public information. … All tables were produced from this altered file. (U.S. Census Bureau 2001)

The point of swapping is to introduce uncertainty. Swapping ensures that the information provided by a respondent cannot be confidently linked to a particular identified individual. “Because of data swapping, users should not assumethattableswithcellshavingavalueofoneortworevealinformation about specific individuals” (U.S. Census Bureau 2003, 8–3).

For the long-form sample questionnaire, the 1990 census employed an additional confidentiality measure: blanking and imputation. For one household in each block group, some specific values were blanked out and imputed with values “donated” from similar individuals. The imputed data were used to produce both the tabulations of long-form data and the 1990 PUMS, providing an additional layer of protection against disclosure.

Leading up to the 2000 Census, some Census Bureau analysts became concerned about potential disclosure risks, especially for microdata (Zayatz 1999). They argued that increasing availability of digital data—such as voter registration lists and commercial databases—together with declining costs of computing, had increased the risks of reidentification. In a reidentification attack, an external dataset that identifies particular individuals is matched to the census microdata file. Although the use of sampling, swapping, and imputation made it impossible to identify anyone’s census responses with certainty, the Census Bureau nevertheless wanted to further strengthen privacy protections. Accordingly, the Census Bureau proposed to create microdata samples with far less detail than had been available in previous census years. For example, instead of the 298 specific countries of birth identified in the 1990 census, the Bureau proposed to provide information on only the major continent of birth (Robbin 2001; Ruggles 2000; Ruggles et al. 2000).

After extensive feedback from the user community, the Census Bureau modified its plans (Robbin 2001). All variable categories representing fewer than 10,000 persons in the general population were combined into larger categories. The swapping procedure was modified to focus on cases with the highest risks of disclosure, especially persons or households that were unique within a small area. In addition, the Census Bureau used a perturbation procedure to randomly modify some ages (Alexander, Davern, and Stevenson 2010; Cleveland et al. 2012).^4^

Similar procedures were subsequently used for the American Community Survey (ACS), which replaced the long form of the census after 2000. Guided by empirical reidentification experiments, the Census Bureau continued to refine disclosure controls to further reduce the risk of reidentification (McKenna 2019). These include altering the swapping routine, better identifying households that could pose a reidentification risk, and slightly increasing the percentage of households that are swapped (Lauger, Wisniewski, and McKenna 2014).

The Census Bureau disclosure control strategy from 1970 to 2010 focused on ensuring that the identity of respondents—such as their name, address, or Social Security number—cannot be inferred from census publications. The Census Bureau implemented targeted strategies to prevent reidentification attacks so that an outside adversary cannot positively identify which person provided a particular response. The protections in place during this period—sampling, swapping, suppression of geographic information and extreme values, imputation, and perturbation—worked extremely well to meet this standard. Indeed, there is not a single documented case of anyone outside the Census Bureau revealing responses of a particular identified person using data from the decennial census.

For the 2020 Census, the U.S. Census Bureau implemented new disclosure control strategies that mark a “sea change for the way that official statistics are produced and published” (Garfinkel, Abowd, and Powazek 2018, 136). The new procedures did not arise in response to public concerns about disclosure risk. There was little public anxiety about confidentiality leading up to the 2020 census; rather, the new confidentiality procedures emerged from concerns among Census Bureau staff.

The new approach adds random error to every population count the agency produces for geographic units below the state level. Over the past several years, multiple outside investigators have examined the implications of the Census Bureau’s new disclosure control algorithms for the accuracy and reliability of the data (e.g., Santos-Lozada et al. 2020; Van Riper, Kugler, and Ruggles 2020; Hauer and Santos-Lozada 2021; Kenny et al. 2021; Winkler et al. 2022; Mueller and Santos-Lozada 2022; Asquith et al. 2022). These studies suggest that the new methods introduce systematic biases that reduce the usability of census data for social, economic, and health research and have the potential to compromise the integrity of basic demographic measures.

The Census Bureau argues that the new disclosure controls are needed because of the threat of “database reconstruction,” a procedure for inferring individual-level characteristics from tabular data (Garfinkel, Abowd, and Martindale 2018). To demonstrate the disclosure risk, the Census Bureau conducted an experiment to reconstruct five characteristics (census block, age, sex, race, and whether Hispanic) for the entire 2010 population using only published census tables. The experiment found that most of the reconstructed cases did not match anyone in the real population, and the Census Bureau acknowledged there were no means for an outsider to determine which of the reconstructed cases were correct (Jarmin 2019).

Several analysts have published critiques of the Census Bureau Database Reconstruction Experiment (Ruggles et al. 2019; Ruggles and Van Riper 2022; Muralidhar 2022; Francis 2022; Muralidhar and Domingo-Ferrer 2023a, 2023b; Sanchez, Domingo-Ferrer, and Muralidhar 2023). They contend that the experiment was neither a reconstruction nor a reidentification as those terms have been previously understood in the literature. The analyses have demonstrated that most of the matches the Census Bureau found between the reconstructed data and the original census would be expected purely by chance (Ruggles and Van Riper 2022). Moreover, there are many billions of reconstructions consistent with the published tabulations, and they are all equally likely (Muralidhar 2022). There is a consensus among the critics that the Census Bureau Experiment failed to demonstrate a realistic threat to confidentiality. At this writing, the Census Bureau has not responded to these reports.

## Discussion

Americans have been expressing concerns about census privacy since the first census in 1790. In response to public concerns about privacy and government overreach, Census officials began making promises of confidentiality in 1840. Those promises expanded greatly over the next two centuries, but this had little impact on privacy concerns. Confidentiality promises did not address privacy concerns because the public was not concerned about disclosure, but rather about government intrusiveness.

It is an article of faith at the Census Bureau and among privacy experts that strong confidentiality guarantees are essential for maximizing response rates to censuses and surveys. There is, however, little evidence to support this conclusion. A Census Bureau study in the 1990s determined that promises of confidentiality had no significant effect on response rates (Dillman et al. 1996). Moreover, experimental studies have consistently found that assurances of confidentiality can actually *increase* concerns about confidentiality and *reduce* response rates to surveys (Berman, McCombs, and Boruch 1977; Frey 1986; Reamer 1979; Singer, Hippier, and Schwartz 1992).

The three waves of public anxiety about census privacy in 1940, 1970, and 2000 appear to have had modest consequences for public cooperation with the census. Net census undercount dropped steadily from 5.3 percent in 1930 to 0.1 percent in 2000, except for an uptick in 1990 (King and Magnuson 1995; Hacker 2013; O’Hare 2014). The level of public worry about census privacy seems to have dropped in recent decades. Since 1980, the Census Bureau has sponsored a series of surveys preceding each census to gauge attitudes towards the census. In 1980, 1990, 2000, and 2010 the surveys asked whether respondents agreed with the statement that “the census is an invasion of privacy.” Between 1980 and 2010, the percentage agreeing dropped from 23.6 percent to 16.0 percent (Moore 1982; Faye, Bates, and Moore 1991; Singer, Hoewyk, and Tourangeau 2001; Conrey, ZuWallack, and Locke 2012). The series of data points suggests a modest downward trend in public concern about census privacy after 1980, interrupted briefly in 2000 following the extensive news coverage about the intrusiveness of the census (Martin 2007).^5^

Even as the overall coverage of the Census improved over time, self-response rates dropped from almost 80 percent in 1970 to 60.5 percent in 2020 (O’Hare, Brumfield, and Lee 2020). Given that the level of public concern about census privacy also appears to have fallen, privacy fears cannot plausibly explain the decline in response rates. In any case, privacy concerns are only weakly associated with self-response rates. Census Bureau studies in 1990 and 2000 found that people who were concerned about invasion of privacy or about government misuse of census data were statistically significantly less likely to mail back their census form, but the effect was very small (Singer, Mathiowetz, and Couper 1993; Singer, Van Hoewyk, and Neugebauer 2003).

In 2017, the U.S. Commission for Evidence-Based Policymaking made a compelling case for the social benefit of broadening access to data from federal agencies (Abraham et al. 2018). That report became the basis for the Foundations for Evidence-Based Policymaking Act of 2018 (2019), which requires each agency to develop open data plans and make federal data publicly available by default. In the ensuing years, however, growing concerns about confidentiality have led to *reductions* in access to reliable census data.

Over the six decades since detailed electronic census data have been available, there is no evidence that any person has been harmed through inadvertent disclosure of private information in a Census Bureau statistical publication. Nor is there evidence that the public is greatly concerned about the potential for such inadvertent disclosure, nor that fear of public disclosure has had significant impact on public cooperation with the census. Until the recent sea change in policy, Census Bureau disclosure protection had minimal impact on access to reliable data. Accurate data are essential for planning and policymaking as well as for social, economic, and health research. Reducing data quality may backfire as it will degrade the rationale for producing the census in the first place. As Hotz et al. (2022) have argued, statistical agencies should therefore carefully weigh realistic measures of disclosure risk against the impact of heavy-handed disclosure control on data utility.

## Footnotes

1 Permission to provide individual data at the discretion of the Superintendent/Director of the Census stood until Title 13, Section 8 was passed in 1976 (Bohme and Pemberton 1991, 6; Eckler 1972, 165).

2 Some cartoons gave a more positive reaction to the new questions. The housing census asked a new question on “principal refrigeration equipment” and a New Yorker cartoon depicted a woman directing the salesman to “be sure to deliver the new refrigerator in time for the census” (“And be sure to deliver it in time for the census,” 1940, 14). A New York Times cartoon suggested that Americans were happy to crowd into a picture and answer all kinds of questions from mortgage status to unemployment (“An Intimate Portrait of a Famous Family,” 1940, 72).

3 Nonresponses to the income question estimated from Ruggles et al. (2023).

4 Unfortunately, the age perturbation was poorly executed, and the sex ratios of personsaged65andolderwerebadlyskewed in both the Census 2000 microdata and the early ACS microdata. The Census Bureau released new files in 2009 and 2010 that correct the sex ratios, but the methods used were not disclosed, and by some measures—such as the age intervals between spouses—the systematic error introduced by perturbation increased (Cleveland et al. 2012).

5 Hillygus et al. (2006, 86) analyzed a series of surveys taken during 2000 as the census privacy controversy unfolded, and they concluded that the debate “had a large and direct impact on cooperation levels in the 2000 census.”
